# Recent progress in the discovery of small molecules for the treatment of amyotrophic lateral sclerosis (ALS)

**DOI:** 10.3762/bjoc.9.82

**Published:** 2013-04-15

**Authors:** Allison S Limpert, Margrith E Mattmann, Nicholas D P Cosford

**Affiliations:** 1 Apoptosis and Cell Death Research Program, Sanford-Burnham Medical Research Institute, 10901 N. Torrey Pines Road, La Jolla, California 92037, United States

**Keywords:** amyotrophic lateral sclerosis (ALS), copper/zinc (Cu-Zn) superoxide dismutase 1 (SOD1), glutamate toxicity, neurodegeneration, oxidative stress

## Abstract

Amyotrophic lateral sclerosis (ALS) is a fatal neurodegenerative disorder with few therapeutic options. While several gene mutations have been implicated in ALS, the exact cause of neuronal dysfunction is unknown and motor neurons of affected individuals display numerous cellular abnormalities. Ongoing efforts to develop novel ALS treatments involve the identification of small molecules targeting specific mechanisms of neuronal pathology, including glutamate excitotoxicity, mutant protein aggregation, endoplasmic reticulum (ER) stress, loss of trophic factors, oxidative stress, or neuroinflammation. Herein, we review recent advances in the discovery and preclinical characterization of lead compounds that may ultimately provide novel drugs to treat patients suffering from ALS.

## Introduction

Amyotrophic lateral sclerosis (ALS), also known as Lou Gehrig’s disease, is a progressive neurodegenerative disease that leads to the dysfunction and death of motor neurons in both the motor cortex and spinal cord. This adult-onset disorder leads to paralysis and eventual death, most commonly by asphyxiation. Symptoms typically include muscle weakness and wasting, cramps, poor reflexes, twitching, and speech problems [1]. Few treatment options exist for this fatal disease, which typically results in death within 2–5 years of diagnosis [2]. Currently, riluzole (**1**), a compound which reduces glutamate excitotoxicity, is the only FDA approved drug for the treatment of ALS. However, its benefits are meager, as it has no effects on disease symptoms and only extends lifespan for an average of 2–3 months [3].

World-wide, the incidence of ALS is 1–2 in 100,000 individuals with about 90% of cases being sporadic (sALS) and 10% of all cases characterized as familial (fALS) [4]. Several gene mutations have been identified that contribute to this disorder with 20% of fALS cases being linked to mutations in the copper/zinc (Cu-Zn) superoxide dismutase 1 (SOD1) gene [4]. Many cellular pathologies have been characterized in ALS, including, but not limited to glutamate toxicity, protein misfolding and aggregation, endoplasmic reticulum (ER) stress, loss of trophic factors, oxidative stress, inflammation, disrupted protein trafficking, and mitochondrial dysfunction [5]. Therapeutic development has been based around the targeting of these mechanisms of cellular dysfunction.

**Figure 1:**
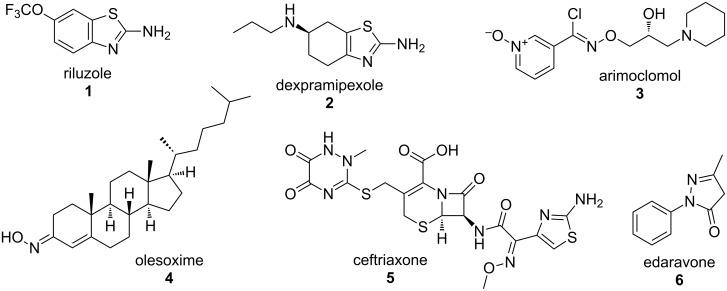
FDA-approved riluzole (**1**) and other ALS drugs currently in phase III clinical trials (**2**–**6**).

Currently, several drugs are in phase III clinical trials for the treatment of ALS (comprehensively reviewed in Glicksman, 2012 [3] and Dunkel et al., 2012 [5]). These drugs include dexpramipexole (**2**), a mitochondrial stabilizer; arimoclomol (**3**), a heat-shock protein (hsp) coinducer; olesoxime (**4**), a mitochondrial pore modulator; ceftriaxone (**5**), an inducer of the glial glutamate transporter (GLT1, EAAT2); and edaravone (**6**), a free-radical scavenging agent (Figure 1). Our focus in this review is to primarily highlight novel small molecules in the discovery and preclinical development stages for the treatment of ALS and to discuss their relevance in the context of current advances in the field.

## Review

### Animal models of ALS

The discovery of genetic mutations in fALS has led to the development of transgenic mouse models and cell-culture systems to study this disorder. The most common of these mouse models carries the SOD1 G93A mutation where glycine is substituted for alanine at position 93 in the superoxide dismutase 1 protein [6–7]. Other related mutations in SOD1 include H46R, A4V, and G85R. These mutations are not believed to reduce the function of the SOD1 protein; however, they have been hypothesized to cause selective motor-neuron death through a toxic gain of function [8]. Mutant isoforms of the SOD1 protein form intercellular aggregates leading to disruption of the proteasome, ER stress, mitochondrial dysfunction, and other cellular deficits and damage [8]. SOD1 mutant mice display prominent motor-neuron degeneration and have many of the hallmarks of human ALS, including progressive hind-limb weakness, increasing weight loss, and eventual paralysis and death [8]. Recently, additional genes have been implicated in ALS, such as TARDBP, which encodes for the trans-activating response (TAR) DNA-binding protein 43 (TDP-43), FUS/TLS*,* which encodes the RNA-binding protein fused in sarcoma, and VAPB, a gene encoding the vesicle-associated membrane-protein-associated protein B/C, and animal models based on mutations in these genes have been developed [8].

Despite obvious parallels with human ALS, to date these transgenic mouse models have proven ineffective in producing potential drug therapies [6]. Many drugs that show efficacy in mouse models have been unproductive in patient trials. Furthermore, riluzole [6-(trifluoromethoxy)-2-aminobenzothiazole], the only FDA approved compound for ALS, produced only very modest effects on disease progression in SOD1 G93A transgenic mice when administered prior to symptom onset [4]. These results highlight the limitations of these animal models in drug development and question how effective these models are in therapeutic discovery.

### Reduction in glutamate toxicity

**Figure 2:**
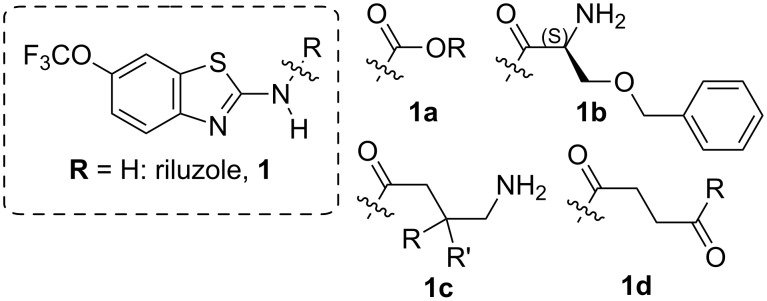
Riluzole (left) and prodrugs developed by McDonnell et al. [11].

Riluzole (**1**), the only currently approved treatment for attenuating disease progression in ALS patients, both inhibits the release of glutamate and noncompetitively inhibits postsynaptic NMDA and AMPA receptors [6]. However, riluzole demonstrates variable drug exposure in addition to highly differential serum concentrations among ALS patients following oral administration [9]. This variability correlates with the heterogeneous patient expression of the cytochrome P450 (CYP) isoform CYP1A2, which provides the primary mechanism of riluzole metabolism [10–11]. Given this variability in metabolism within the patient population, recent studies have focused around creating riluzole prodrugs that would exhibit higher stability in vivo [11]. For example, McDonnell et al. [11], identified and evaluated a group of 23 riluzole prodrugs for their potential use in the treatment of glutamate toxicity in ALS and other disorders. Potential drug candidates were prepared through the conversion of the exocyclic amine to single alpha amides, carbamates, succinamides, or amide linkages from γ-aminobutyric acids (Figure 2). It is expected that these compounds would be cleaved by amidases or esterases found in plasma to generate riluzole. The stability of these analogues was tested in simulated gastric fluid, simulated intestinal fluid, and in liver microsomes to determine whether the drugs would enter the plasma intact. Further, the liberation of riluzole from the prodrugs was evaluated in plasma. One compound, an *O*-benzylserine derivative of riluzole (Figure 2, **1b**), was identified as a candidate prodrug appropriate for in vivo testing, due to its stability in in vitro intestinal and microsomal assays and its ability to withstand metabolism by CYP1A2 [11]. Further development of this prodrug may allow for consistent riluzole plasma levels and thus more efficacious treatment among ALS patients.

**Figure 3:**
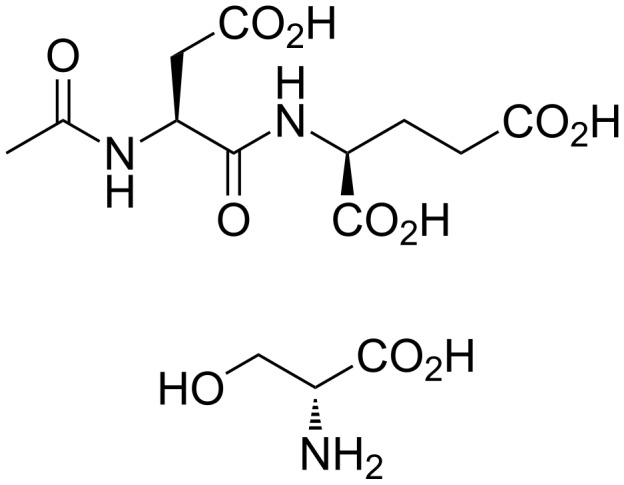
Neurotransmitters *N*-acetyl-aspartyl glutamate (NAAG, top) and D-serine (bottom).

The modest success of riluzole in ALS treatment and the role of glutamate excitotoxicity in numerous disease states have motivated further drug development focused on the modulation of glutamate signaling. In particular, evidence for an essential role of glutamate toxicity in ALS has come from the analysis of cerebrospinal fluid (CSF) from ALS patients, which shows a three-fold increase in glutamate and *N*-acetyl-aspartyl glutamate (NAAG, Figure 3) levels relative to controls [12–13]. Furthermore, exposure of CSF extracted from ALS patients kills healthy motor neurons in culture [14]. Together, these data point to an excess of glutamatergic signaling in ALS and suggest that decreasing glutamate levels may have therapeutic benefits in ALS patients. The actions of glutamate, the primary excitatory neurotransmitter in the nervous system, are terminated by the uptake of glutamate away from the synapse by numerous glutamate transporters [15]. In particular, the Na^+^-dependent excitatory amino acid transporter 2 (EAAT2), which is present on glial cells surrounding the neuronal synapse [12], is predominantly involved in the clearance of glutamate from the synapse. Thus, activators of EAAT2 have the potential to reduce glutamate toxicity in vivo and attenuate the disease progression of ALS.

As EAAT2 expression is highly regulated at the translational level, one strategy for increasing EAAT2 activity is to use small molecules to increase the translation of EAAT2 mRNA [6]. This strategy, employed by Colton et al. [6], prompted the screening of a library of 140,000 compounds by using an ELISA-based assay for EAAT2 protein expression. This screen resulted in 293 hits for compounds increasing EAAT2 expression. Of these, three were selected as lead compounds for further optimization based on their potency and lack of cellular toxicity, although it should be noted that the structures of the hits were not disclosed [6]. Additionally, the EAAT2 protein induced by these lead compounds was found to be functional and exhibit appropriate cellular localization [6].

**Figure 4:**
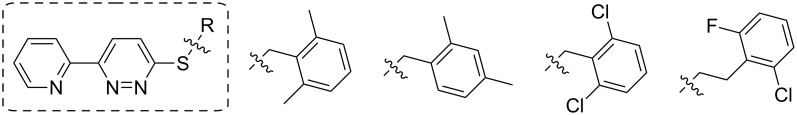
Thiopyridazines developed to increase EAAT2 protein levels.

Using these identified lead compounds, Xing et al. [16] performed chemical optimization to develop additional analogues for potential use as therapeutic agents. Structure–activity relationship (SAR) studies revealed that the thioether and pyridazine moieties were essential molecular components for increasing EAAT2 protein levels [16]. Of the analogues developed, several thiopyridazine derivatives (Figure 4) were found to increase EAAT2 levels greater than six-fold over endogenous levels in primary astrocyte (PA)-EAAT2 cells (an astrocyte cell line stably expressing mRNA for EAAT2) at concentrations of less than 5 µM. Additionally, one derivative was found to increase EAAT2 levels 3–4-fold at only 0.5 µM [16]. These compounds will prove useful for evaluating the potential of EAAT2 activators in animal models of ALS and in the study of other diseases where glutamate toxicity plays an essential role.

In addition to dysregulation of glutamate levels in ALS patients, recent studies have also detected elevated levels of D-serine (Figure 3), an activator/co-agonist of the *N*-methyl-D-aspartate (NMDA) ionotropic glutamate receptor, in the spinal cord of both ALS patients and transgenic mice carrying the SOD1 G93A mutation [17–18]. This increase in D-serine corresponded with a reduction of D-amino acid oxidase (DAO) in SOD1 mutant mice, the enzyme responsible for the metabolism of D-amino acids including D-serine. Interestingly, a new mutation in the D-amino acid oxidase (DAO) gene has been recently characterized to contribute to fALS [19]. This R199W DAO mutation inhibits the function of DAO, increases ubiquitin-containing aggregates, and reduces cell viability when expressed in neuroblastoma-spinal cord (NSC)-34 cells, a motor neuron cell line [19]. Since D-serine serves as a co-agonist at the glycine site of the NMDA glutamate receptor, increases in D-serine are likely to contribute to glutamate excitotoxicity in ALS patients. These data suggest that reducing D-serine levels through activation of DAO or reduction of serine racemase (SR), the enzyme responsible for D-serine synthesis, may be therapeutically beneficial [20]. Furthermore, drugs modulating NMDA receptor signaling may also prove beneficial to ALS treatment.

The contribution of improper glutamate regulation to ALS pathology is further highlighted by studies demonstrating abnormal metabotrophic glutamate (mGlu) receptor expression in ALS patients. Elevated levels of Group I, II, and III mGlu receptors have been reported in astrocytes of ALS patients, while a decrease in the levels of Group II mGlu receptors has been detected in neurons of the spinal cord in these patients [21]. Furthermore, T-lymphocytes in ALS patients display reduced mGlu_2_ receptor levels as compared to controls [22]. These data substantiate the role of glutamatergic dysfunction in ALS and indicate that non-neuronal cells may be affected [22].

### Targeting SOD1 mutations

**Figure 5:**
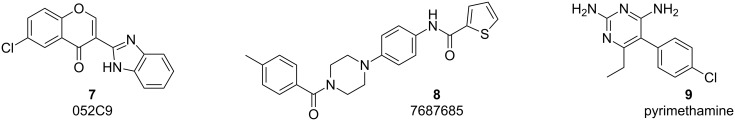
Compounds shown to reduce SOD1 expression.

Due to the role of SOD1 mutations in fALS and the reproduction of human ALS pathology in mouse models carrying mutant SOD1 genes, one strategy to attenuate ALS pathology is to develop small molecules that reduce SOD1 protein levels. Support for targeting SOD1 protein expression has come from animal studies demonstrating that the reduction of SOD1 protein levels in motor neurons causes these cells to become resistant to ALS-induced cellular death [23]. In order to identify small molecules that downregulate the transcription of SOD1, Murakami et al. [24] developed a high-throughput screening assay using an H4 human astrocytoma cell line expressing a SOD1 luciferase reporter construct. Following a screen of a library of 9600 small molecules, 325 compounds were identified as hits, with 2 compounds demonstrating selectivity in downregulating SOD1 protein levels without discernible cellular toxicity following secondary assays [24]. One of these compounds was chosen for further analysis due to its considerably lower 50% effective concentration (EC_50_). Interestingly, this selected hit compound, 3-(1*H*-benzo[*d*]imidazol-2-yl)-6-chloro-4*H*-chromen-4-one (052C9, **7**; Figure 5), was found to reduce phosphorylation of the transcription factor Nrf2, a known activator of cellular stress genes as well as an upregulator of SOD1 transcription [24].

A similar high-throughput screen was performed by Wright et al. [25], who assayed 30,000 small molecules for SOD1 transcriptional repression by employing a PC12 (phenochromacytoma) cell line stably expressing the human SOD1 promoter flanked by green fluorescent protein (GFP) [25]. This screening strategy identified 20 compound hits, for which the activity was confirmed through secondary assays and analyzed for cytotoxicity. Compound 7687685 (**8**; Figure 5) was demonstrated to both reduce endogenous SOD1 protein levels in human cells and also repress several other genes implicated in ALS including FUS and TARDBP [25]. However, in in vivo studies performed in SOD1 G93A transgenic mice, compound **8** exhibited only a small (5%) reduction of SOD1 protein levels in spinal-cord extracts. Due to the toxicity of the compound when administered in higher doses, compound **8** is unlikely to be useful for the treatment of ALS patients, although this screening strategy may prove relevant for the development of further small molecule inhibitors [25].

Conflicting data have arisen surrounding the ability of the anti-malarial compound pyrimethamine (**9**; Figure 5) to reduce SOD1 protein levels. Lange et al. [26] identified a dose-dependent reduction in SOD1 expression in cultured human cells and performed a phase I pilot study in 16 ALS patients. This study analyzed blood and CSF samples of patients treated with the drug for 18 weeks and determined that SOD1 levels were significantly reduced in CSF and in leukocytes of these individuals [26]. However, Wright et al. [27] were unable to confirm these results in either cultured cells or in mice treated with pyrimethamine. In contrast, these studies found that the concentrations of pyrimethamine required to reduce transcriptional activity from the SOD1 promoter by 42% caused a 68% reduction in cellular viability, thus leading to the conclusion that the reduction in SOD1 levels was due to nonspecific cytotoxicity. In other cell types, as well as in animal studies, pyrimethamine was unable to reduce SOD1 protein levels as compared to controls [27]. These conflicting results are likely due to differences in how SOD1 protein levels were assessed as well as due to differences between human and mouse fluid samples. Further studies will be required to determine the effects of pyrimethamine treatment on SOD1 protein expression and more importantly assess whether or not it is able to attenuate ALS pathology.

**Figure 6:**
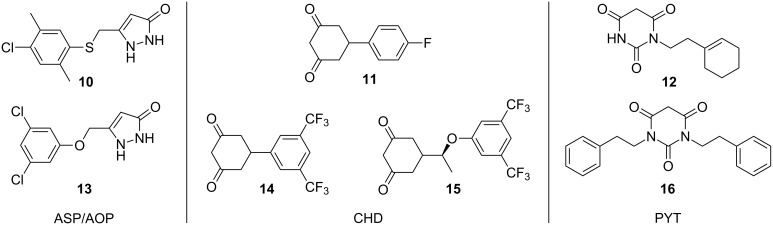
Families of compounds (named in italics) capable of reducing SOD1-induced cellular toxicity and mutant SOD1 protein aggregation. Top: selected compounds identified in high-throughput screening. Bottom: advanced compounds.

Mutations in SOD1 lead to cellular toxicity not through loss of function of the SOD1 protein, but rather through a toxic gain of function, whereby SOD1 mutants aggregate in intercellular inclusions leading to cellular dysfunction. Due to this mechanism of SOD1-induced cellular death, compounds that reduce the aggregation of SOD1 protein may be able to protect cells from damage. Benmohamed et al. [28] developed a screening strategy to analyze the ability of small molecules to reduce mutant SOD1 aggregates in a cell-culture model [28]. Using PC12 cells transfected with an inducible SOD1 G93A construct [29], a library of over 50,000 small-molecule compounds was initially screened for the ability to enhance cellular viability in the presence of the mutant SOD1 protein [28]. Hits from this screen were then subjected to several counter screens including a mutant SOD1 aggregation assay that utilized a cell line expressing a SOD1 G85R mutant protein [29] coupled to yellow fluorescent protein (YFP). Following incubation with the selected compounds, the SOD1 G85R YFP cells were imaged using a high throughput fluorescent microscopy system and analyzed for the number of SOD1 aggregates per cell [28]. This screening strategy, combined with chemoinformatic methodologies used to cluster structurally similar compounds, allowed the researchers to identify three distinct chemical series that were selected for optimization based on their ability to reduce both cellular toxicity and mutant SOD1 protein aggregation: arylsulfanyl pyrazolones (ASP, **10**), cyclohexane-1,3-diones (CHD, **11**), and pyrimidine 2,4,6-triones (PYT, **12**; Figure 6).

The ASP derivatives were subjected to structural optimization and the resulting compounds were then evaluated in pharmacokinetic (PK) assays. Two ASP compounds, which demonstrated activity in cell viability and SOD1 aggregation assays, were found to have low microsomal stability and poor brain accumulation, respectively [30]. Metabolic profiling and further chemical modification were performed to increase the stability and potency of ASP derivatives, and this ultimately led to replacement of the thioether with an ether linkage and the identification of a new aryloxanyl pyrazolone (AOP) scaffold exemplified by compound **13** [31]. The new AOP analogues were optimized and tested in cell-viability assays in primary neurons, as well as aggregation assays in SOD1 mutant-expressing cells. Compound **13** displayed high activity in these assays as well as a promising pharmacokinetic profile including good penetration of the blood–brain barrier (BBB) and was further tested in a SOD1 G93A transgenic mouse model. Mutant mice treated with compound **13** by intraperitoneal (i.p.) injection at 20 mg/kg daily, starting at 6 weeks of age, displayed a 13.3% increase in lifespan as compared to controls [31], suggesting that the AOP scaffold is potentially suitable for therapeutic development for the treatment of ALS. Several important findings in the development of pyrazolone compounds included the identification of an *N*1-benzyl substituted pyrazolone, which displayed enhanced potency along with the discovery that the *N*2-H group participates in hydrogen-bond-donating interactions with the biological target [32].

SAR around the CHD scaffold **11** determined that 3,5-ditrifluoromethyl analogue **14** had the highest potency of these derivatives. Additionally, **14** possessed favorable PK, demonstrating high plasma stability and oral bioavailability, as well as high brain accumulation [33]. Due to its advantageous pharmacological properties, **14** was tested in SOD1 G93A transgenic mice to determine whether it was able to extend lifespan and alleviate symptoms in a mouse model of ALS. However, this compound demonstrated no therapeutic benefit. Additional studies demonstrated that **14** exhibited poor activity in primary cortical neurons due to low penetration of neuronal cells [33]. Further SAR around this series led to new chiral CHD analogues, such as compound **15** (Figure 6), with higher neuronal permeability and potency. Additionally, these compounds were found to be active in the cytotoxicity screen performed in SOD1 G93A-PC12 cells and displayed favorable PK profiles. Importantly, compound **15** exhibited a 90% increase in activity in primary cortical neurons [34]. Because of these favorable properties, this analogue was tested in SOD1 G93A transgenic mice that were treated daily by i.p. administration with 30 mg/kg of CHD derivative compound **15** starting at 6 weeks (prior to symptom presentation). A 13% increase in lifespan was observed in treated animals as compared to controls [34].

SAR studies with PYT scaffold **12** were also successful in identifying an analogue with properties suitable for use as a novel therapeutic for ALS. Modifications to the PYT backbone were made and subsequent compounds were tested in both the previously described cytotoxicity assay as well as the SOD1 aggregation assay. Compound **16** was found to be highly active in both of these assays and additionally demonstrated high potency and low toxicity, as well as excellent solubility and plasma stability [35]. Further studies indicated that compound **16** was able to cross the BBB and exhibited good oral bioavailability [35].

An alternative strategy to prevent the aggregation of SOD1 was employed by Ray et al. [36], who designed small molecules to stabilize the SOD1 native dimer, theorizing that SOD1 monomerization was required for aggregate formation [36]. Examination of the mutant SOD1 A4V dimer interface detected hydrophobic cavities that could be filled to enhance protein stability. When these cavities were filled by genetic mutagenesis of the SOD1 protein, enhanced dimer stability was detected [36]. An in silico screen was performed to identify compounds with the potential to bind at the dimer interface and the top 100 hits were screened in an SOD1 A4V aggregation assay. Fifteen compounds inhibited the aggregation of SOD1 A4V proteins and were successfully found to prevent the aggregation of other SOD1 mutants, G85R and G93A [36].

**Figure 7:**
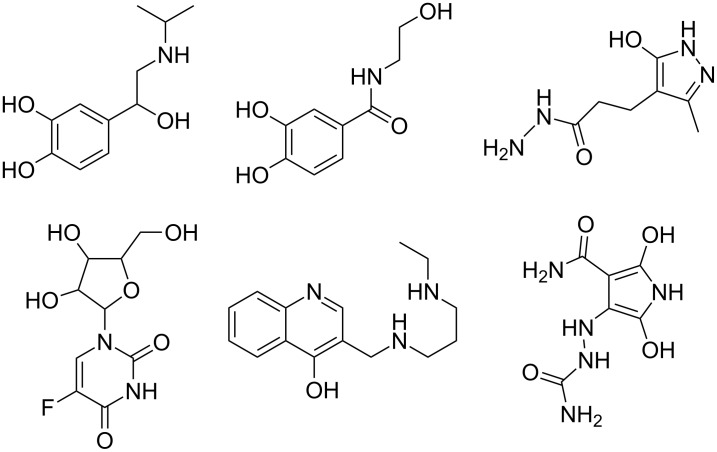
Compounds identified by Nowak and co-workers [37] in silico that selectively bind SOD1 over human plasma and inhibit A4V-SOD1 aggregation.

However, when tested for SOD1 protein binding in the presence of human blood plasma, these compounds performed poorly, binding with higher affinity to blood proteins than to SOD1, suggesting that these compounds may have significant off-target activity [37]. Docking calculations were performed to model the inhibitors at the dimer interface and a database of small molecules was screened to identify molecules that satisfied the docking constraints [37]. Twenty new compounds were identified and analyzed for inhibition of SOD1 A4V aggregation as well as binding to SOD1 in the presence of human plasma. Six of these compounds (Figure 7) tested positively in these assays [37], indicating that they may be excellent starting points for therapeutic development for ALS.

### Targeting TDP-43

**Figure 8:**
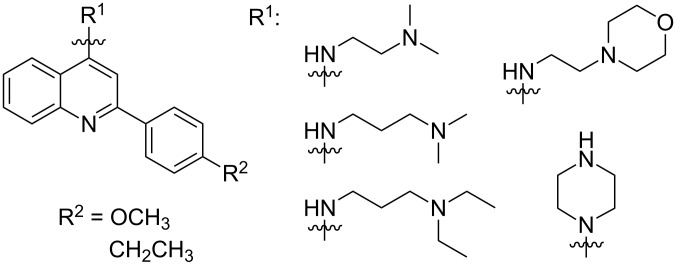
4-Aminoquinolines developed by Cassel and co-workers [43] for disruption of oligonucleotide/TDP-43 binding.

While SOD1 mutations are frequently studied, these mutations account for about 20% of familial ALS and only 2–3% of all ALS cases [2]. Recent studies have focused on creating small molecules that target other mutant proteins associated with ALS. Trans-activating response (TAR) DNA-binding protein 43 (TDP-43) is a nucleotide-binding protein important for gene transcription and mRNA splicing, transport, and stabilization [38]. Mutations in the TARDBP gene, which encodes TDP-43, are responsible for up to 6.5% of fALS [1]. In the neurons of ALS patients, TDP-43 protein is decreased in the nucleus and accumulates in cytoplasmic inclusions where it can sequester cytoplasmic RNAs in stress granules [39–41]. One approach to alleviate the pathology caused by mutant TDP-43 is to identify small molecules that inhibit the binding of TDP-43 to nucleotides. Cassel et al. [42] developed a high-throughput screening assay whereby TDP-43 nucleotide binding could be assessed. A screen of 7360 compounds yielded a series of small molecules that disrupt oligonucleotide binding to TDP-43 protein [42]. Later, this series of 4-aminoquinoline derivatives (Figure 8) was tested for its ability to regulate TDP-43 [43].

TDP-43 expression levels must be appropriately regulated by the cell as either overexpression or deletion of TDP-43 causes cellular death. Caspases 3 and 7 can mediate the reduction of TDP-43 protein levels through cleavage of TDP-43 and subsequent clearance of the cleaved products by the proteasome [44]. Cleavage-resistant mutations in TDP-43 are highly toxic to the cell [44]. Cassel et al. [43] hypothesized that the 4-aminoquinoline series identified in their HTS screen may increase the rate of caspase cleavage of TDP-43 and thus affect its cellular accumulation. In this study, several 4-aminoquinoline derivatives (Figure 8) were found to bind to TDP-43, decrease its association with oligonucleotides, and increase caspase-mediated cleavage of the protein [43]. Furthermore, treatment of H4 cells with these compounds modestly reduced intercellular levels of TDP-43 [43] as well as histone deacetylase 6 (HDAC-6) and autophagy-related protein 7 (ATG-7), proteins known to be regulated by TDP-43 [45–46]. Since reduction of TDP-43 levels in motor neurons may prove to be beneficial to ALS treatment, further development and validation of this series of small molecules may prove valuable for future therapeutic development.

**Figure 9:**
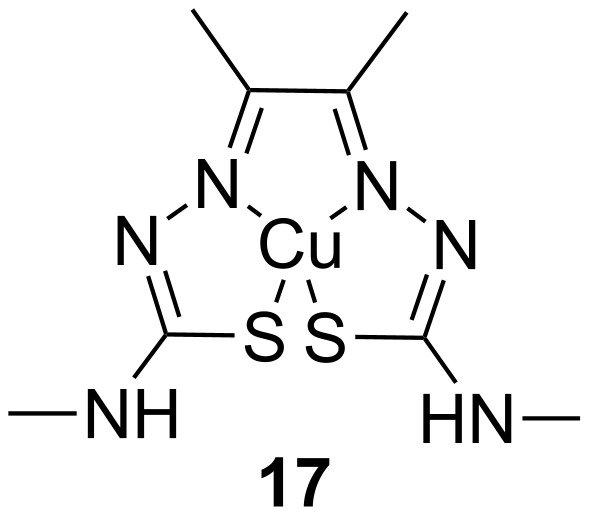
Cu(II)(atsm), an example of a Cu(II)(btsc) copper complex.

Another mechanism to attenuate the toxicity of TDP-43 is to prevent its aggregation into intercellular inclusions. In a study by Parker et al. [38], treatment of SH-SY5Y cells with paraquat to induce cellular stress through mitochondrial inhibition led to the formation of TDP-43 aggregates in the cytoplasm. The formation of TDP-43-containing cellular inclusions was dependent on the activation of stress-induced kinases such as c-Jun N-terminal kinase (JNK). Treatment of cells with bis(thiosemicarbazonato)copper complexes (Cu(II)(btsc)s; Figure 9), reduced stress-induced kinase activity and prevented TDP-43 aggregation [38]. Cu(II)(btsc)s have previously been demonstrated to have neuroprotective effects in mouse models of neurodegeneration [47] and elicited similar results in cells overexpressing TDP-43. These data suggest that Cu(II)(btsc)s, such as compound **17**, may be beneficial in the treatment of ALS by modulating kinase activity and reducing protein aggregation.

**Figure 10:**
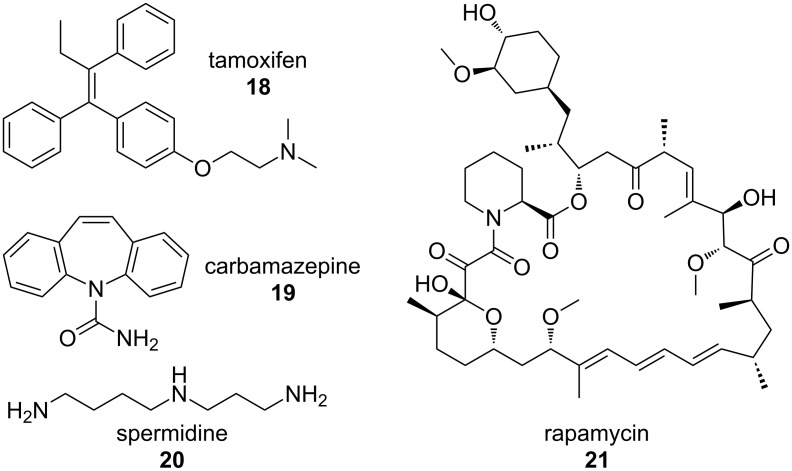
Pharmacological inducers of autophagy.

The removal of dysfunctional proteins and organelles from the cell can occur by the process of autophagy, whereby autophagosomes engulf cellular material, which is then degraded by the lysosome [48]. One strategy to reduce TDP-43-containing cytoplasmic inclusions is to induce autophagy by using known pharmacological activators (Figure 10), such as tamoxifen (**18**), carbamazepine (**19**), spermidine (**20**), or rapamycin (**21**). Studies using these compounds to enhance autophagy in disease models with TDP-43 proteinopathies have discovered a clearance of cytoplasmic TDP-43, as well as a reduction in caspase activation and cellular death corresponding with an upregulation of autophagic markers [48]. Transgenic mice overexpressing TDP-43 in the forebrain display deficiencies in cognition as early as 2 months of age and impairment of motor function at 6 months of age. Treatment of these mice with 10 mg/kg rapamycin by i.p. three times weekly increased their performance in the Morris water maze test at 3 months of age and enhanced rotarod performance at 6 months of age [48]. Together, these data indicate that enhancement of autophagy may reduce cellular death and behavioral dysfunction associated with TDP-43 mutations.

### Modulation of trophic factors

**Figure 11:**
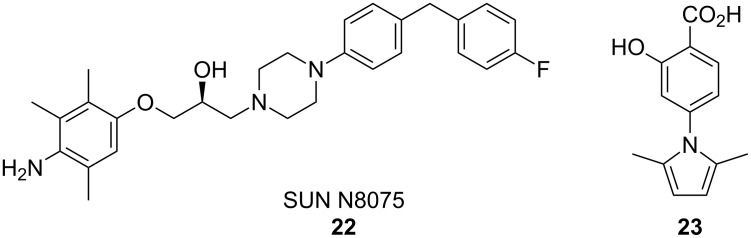
Compounds used to evaluate the effects of trophic factors on ALS disease progression.

One pathological characteristic of ALS is the loss of trophic factors that promote the health and stability of motor neurons. Compounds that increase growth factor-induced neuronal support have been tested in both cellular and mouse models of ALS with moderate success. For example, in a study performed by Shimazawa et al. [49] a small molecule (SUN N8075, **22**, Figure 11), which is currently in clinical trials for the treatment of stroke, protected SH-SY5Y cells against pharmacologically induced ER stress-mediated cell death. Further investigation into the mechanism of action of this compound revealed that **22** potentiated the upregulation of VGF nerve growth factor inducible protein (VGF) in response to cell stress [49]. This potentiation enhanced the activation of cellular survival signals and reduced caspase cleavage. However, siRNA targeting VGF abolished the protective response to **22**, indicating that VGF upregulation was central to the activity of this compound [49].

The importance of VGF in ALS disease progression has been supported by studies of ALS patients, which report a reduction in VGF levels in the CSF of individuals with ALS as compared to control samples from healthy individuals [50]. To determine if **22** could successfully treat ALS symptoms in animal models, transgenic mice carrying the human SOD1 G93A mutation were treated subcutaneously (s.c.) with 30 mg/kg **22** starting at 10 weeks of age and continuing for the lifespan of the animal. Treated animals displayed delayed disease onset and progression as established by rotarod performance. Additionally, animals treated with **22** exhibited a mean increase in lifespan by 10.9%. The effects of **22** were replicated in a transgenic rat model of ALS, where this compound again displayed modest effects on delaying motor function decline and increasing survival [49].

A novel study by Van Hoecke et al. [51] has implicated the Ephrin/Eph system in determining motor neuron susceptibility to degeneration in ALS. Ephrins and their cognate receptors (Eph) are important in nervous-system development where they assist with axonal pathfinding and repulsion. In adults these signaling molecules have been demonstrated to play essential roles in synapse formation and plasticity [52]. In this study, Hoecke et al. [51] used a zebrafish model of ALS to determine modifying factors that could influence disease progression and identified the mammalian EPHA4 gene as a potential disease modifier. To confirm these genetic data, 4-(2,5-dimethyl-1*H*-pyrrol-1-yl)-2-hydroxybenzoic acid (**23**) was used to inhibit EphA4 in mutant zebrafish. This treatment resulted in the rescue of SOD1-induced axonopathy in zebrafish overexpressing a mutant SOD1 isoform. Further studies were performed by genetically reducing EPHA4 gene dosage in mice carrying the hSOD1 G93A mutation. Mice with reduced EphA4 displayed prolonged survival of motor neurons coupled with increased motor performance and lifespan. Rat models of ALS were also employed in this comprehensive study. Rats expressing SOD1 G93A were treated with Epha4 blocking peptide through intracerebroventricular (i.c.v.) injection. Rats injected with blocking peptide exhibited delayed disease onset and prolonged survival. In studies of ALS patients, patients with lower levels of EphA4 protein correlated with later disease onset and individuals carrying mutations in the EPHA4 gene displayed increased survival rates [51]. Finally, studies were performed in zebrafish expressing mutant TDP-43 protein. Inhibition of Epha4 through pharmacologic or genetic methods also rescued axonal deficiencies in this ALS model.

Together these studies suggest that pathways induced by trophic factors that affect growth, development and survival of neuronal cells, are essential components of ALS disease progression. Therapeutics that increase the expression of a prosurvival factor, such as VGF, or inhibit the action of a repressive signaling molecule, such as Epha4, may have a profound effect on patient outcome. Further studies are needed to determine the effects of enhancing VGF or antagonizing Epha4 on other cellular pathways before these treatments have the potential for human testing.

### Neuroprotective compounds

**Figure 12:**
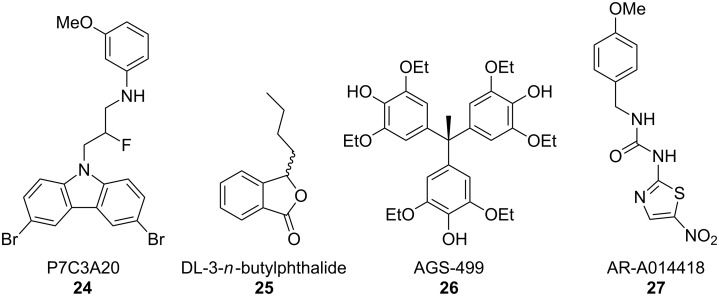
Compounds identified as neuroprotective.

An alternative approach for the treatment of ALS is the use of known neuroprotective or neurogenic compounds (Figure 12). In a screen of a chemically diverse compound library, Pieper et al. identified an aminopropyl carbazole, P7C3, which was found to increase adult hippocampal neurogenesis in an in vivo assay [53]. Further optimization of this compound through structure–activity relationship (SAR) analysis led to the development of an analogue, P7C3A20 (**24**), which has a replacement of the hydroxy group at the chiral center of the linker with a fluorine atom and an addition of a 3-methoxy group to the aniline ring [53]. This compound demonstrated higher potency and was found to protect spinal cord neurons from death in mice expressing the SOD1 G93A mutation. When delivered at the disease onset, **24** demonstrated a reduction in symptom progression as characterized by rotarod tests, and examination of walking gait and stride length [54]. While these data suggest that the use of this compound and its derivatives in treating neurodegenerative disease may be promising, further optimization is required to improve efficacy and solubility as well as reduce toxicity.

Studies using DL-3-*n*-butylphthalide (**25**), a compound approved for use in stroke patients in China, have reported that the treatment of transgenic SOD1 G93A mice can improve motor symptoms and increase lifespan. Oral administration of this compound at 60 mg/kg daily prior to symptom presentation, resulted in no delay in onset of hindlimb weakness, but decreased the progression of motor dysfunction as tested by rotarod [55]. When DL-3-*n*-butylphthalide treatment was initiated following disease onset, SOD1 G93A mice displayed increased survival of motor neurons in the spinal cord and a reduction in astrocyte and microglial activation. Furthermore, transgenic animals treated with this compound increase the expression of the Nrf2 transcription factor, which promotes the expression of anti-inflammatory and prosurvival genes [55].

In a novel approach to inducing neuroprotection, Eitan et al. [56] used triaryl compound 4,4',4''-(ethane-1,1,1-triyl)tris(2,6-diethoxyphenol), designated AGS-499 (**26**), to increase telomerase expression in neuronal cells. Telomerase is a protein complex that maintains the length and integrity of telomeres in developing and dividing cells. In differentiated neurons, telomerase activity is typically absent [57]; however, some studies have indicated that some brain regions maintain active telomerase into adulthood [58–59]. Brain injury results in an increase in telomerase activity and transgenic mice overexpressing telomerase reverse transcriptase (TERT), an essential component of the active telomerase enzyme, displayed a marked resistance to neurotoxicity [60].

Treatment of mice with **26** resulted in an increase in telomerase activity in the forebrain, spinal cord, and brainstem and protected neurons from NMDA-induced toxicity [56]. SOD1 G93A mutant mice injected with **26** displayed a 14.6% reduction in the progression of neurological symptoms as analyzed by limb assessment as well as a 16.4% increase in lifespan [56]. These benefits were mediated by a marked increase in motor neuron survival in the spinal cord. Furthermore, treatment of both rodent motor neuron cultures and human cells with **26** increased TERT levels and protected cells from oxidative stress [56].

An alternative strategy to inducing neuroprotection is to inhibit the signaling molecules that antagonize cellular survival and promote neuron death in disease models. Glycogen synthase kinase-3 (GSK-3) is an essential signaling molecule involved in many cellular processes including glycogen metabolism, cell-cycle regulation, cellular proliferation, and apoptosis. However, studies using tissue samples from ALS patients report that they display elevated GSK-3 levels in the spinal cord [61]. Increased GSK-3 activity has also been reported in the motor neurons of SOD1 G93A mutant mice [62]. Using a GSK-3 inhibitor that crosses the BBB (**27**), Koh et al. [63] examined the effects of reducing GSK-3 activity in mouse models of ALS. SOD1 G93A mice were injected with **27** intraperitoneally at 60 days old. Treated mice displayed delayed symptom onset, reduction in motor deficits as measured by rotarod test, and increased motor neuron survival in the spinal cord. Further investigation determined that the inhibition of GSK-3 in SOD1 G93A mice led to a decrease in cleaved caspase-3 and cytosolic cytochrome c in the spinal cord [63], indicating that the inhibition of GSK3 may be neuroprotective in this disease model. Furthermore, treatment of SOD1 G93A with GSK-3 inhibitors reduced markers of inflammation in the spinal cord [63], suggesting a reduction in glial reactivity.

### Reduction in oxidative stress and inflammation

**Figure 13:**
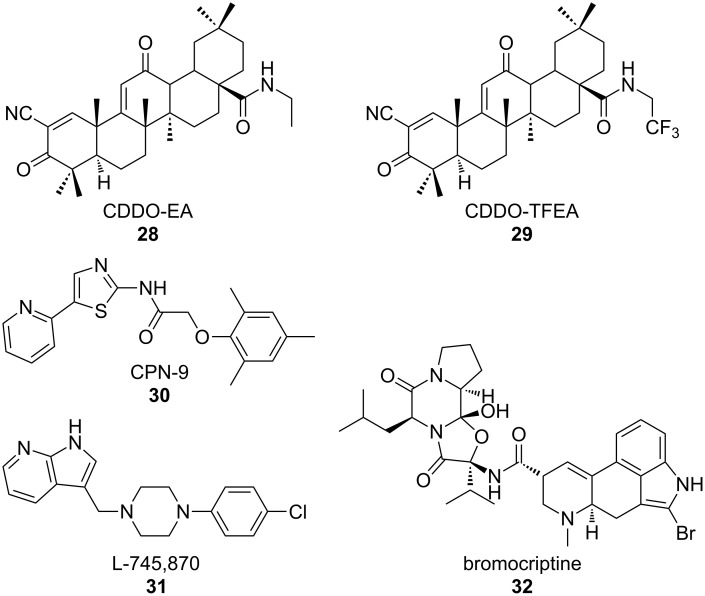
Compounds developed to reduce oxidative stress and inflammation.

Another hallmark of ALS is chronic neuronal exposure to oxidative stress and inflammation and thus several treatment strategies are focused on the reduction of these cellular pathologies. One mechanism to reduce oxidative stress in neurons is to upregulate signaling through the NF-E2-related factor 2/antioxidant response element (Nrf2/ARE) pathway, which is responsible for the upregulation of antioxidant and prosurvival genes. Neymotin et al. [64] tested two related compounds, 2-cyano-3,12-dioxoolean-1,9-dien-28-oic acid-ethylamide (CDDO-EA, **28**, Figure 13) and CDDO-trifluoroethylamide (CDDO-TFEA, **29**), synthetic triterpenoid analogues derived from oleanolic acid [64] for their ability to activate Nrf2/ARE signaling in cell culture and mouse models of ALS. NSC-34 cells expressing SOD1 G93A were treated with **29** and activation of Nrf2 was tested. In response to treatment, the expression of Nrf2 and the Nrf2 regulated genes, NQO-1 (NAD(P)H quinine oxidoreductase), HO-1 (heme oxygenase-1), and glutathione reductase were significantly increased. Furthermore, primary rat neurons treated with **29** displayed an increased nuclear translocation of Nrf2 [64]. Oral treatment of transgenic SOD1 G93A mice with either **28** or **29** resulted in an increase in Nrf2 expression and nuclear localization. The levels of Nrf2-regulated antioxidant genes were also elevated in the spinal cords of treated mice as analyzed by RT-PCR. Importantly, treatment of SOD1 G93A mice with **28** or **29** resulted in reduced weight loss, decreased motor decline and increased lifespan [64].

Using a virtual screening system to discover oxidative-stress-reducing agents, Kanno et al. [65] identified a small molecule, *N*-(5-(2-pyridyl)(1,3-thiazol-2-yl))-2-(2,4,6-trimethylphenoxy)acetamide, termed CPN-9 (**30**). Compound **30** was initially tested for protection against pharmacologically induced oxidative stress and was determined to be highly cytoprotective in HeLa cells. When tested against a variety of cell-stress inducers, **30** only protected against cellular death induced by oxidative-stress pathways [65]. To determine the mechanism by which **30** selectively protects against oxidative damage, the expression of stress-activated proteins HO-1 and p21/CDKN1A was tested. Both stress-induced proteins showed increased expression, and activation of the Nrf2 transcription factor also increased. Compound **30** was demonstrated to induce ARE promoter activity in SH-SY5Y cells by using a luciferase reporter assay [65]. These data demonstrate that **30** confers resistance to oxidative stress by upregulation of the Nrf2/ARE transcriptional pathway.

Due to its success at inhibiting cellular death in cultured cells, **30** was then tested in transgenic mice expressing the hSOD1 H46R mutant gene. Following chronic administration of **30** following symptom onset, disease progression was attenuated as determined by feet clasping and rearing behavior. Mice treated with **30** performed better in functional assays, including rotarod testing and footprint analysis where treated animals showed reduced gait abnormalities. Furthermore, treatment with CPN-9 diminished motor neuron loss in the spinal cord and extended survival following disease onset [65].

Further studies aimed at reducing oxidative stress in ALS models were performed by Tanaka et al. [66], who utilized a dopamine D4 receptor antagonist, L-745,870 (**31**), to selectively inhibit oxidative-stress-induced cell death. Compound **31** was previously determined to upregulate neuronal apoptosis inhibitory protein (NAIP/BIRC1), a cytoprotective protein that ameliorates oxidative-stress-induced cellular death [67]. Intragastric administration of **31** to SOD1 H46R mice, prior to symptom onset, was discovered to delay symptom onset as determined by limb movement, rearing activity, and foot clasping behaviors. Additionally, treatment with **31** delayed weight loss and motor dysfunction as examined by a balance-beam test. Spinal-cord tissue from treated and untreated SOD1 H46R mice was examined for motor-neuron loss and markers of microglial activation. Treated animals displayed reduction in both loss of neurons as well as decreased activation of microglial cells [66]. Additionally, SOD1 H46R mice were treated with **31** following the presentation of disease symptoms. Mice treated with **30** exhibited prolonged survival rates as compared to untreated animals [66].

Additional work from this group identified the dopamine D2 receptor agonist, bromocriptine (**32**), as an NAIP upregulating compound that reduced oxidative stress through the upregulation of antioxidant proteins, such as activating transcription factor 3 (ATF3) and HO-1 [68]. In vivo studies where **32** was administered to SOD1 H46R mice following symptom presentation revealed that **32** delayed disease progression as determined by feet clasping and rearing behaviors as well as improved motor function as analyzed by the balance-beam test, vertical pole test, and footprint analysis. Furthermore, treatment with **32** prolonged the post-onset survival of SOD1 H46R animals [68]. These studies indicate that the attenuation of oxidative-stress pathways through the upregulation of antioxidant genes can reduce disease progression in ALS models.

### Novel mechanisms

**Figure 14:**
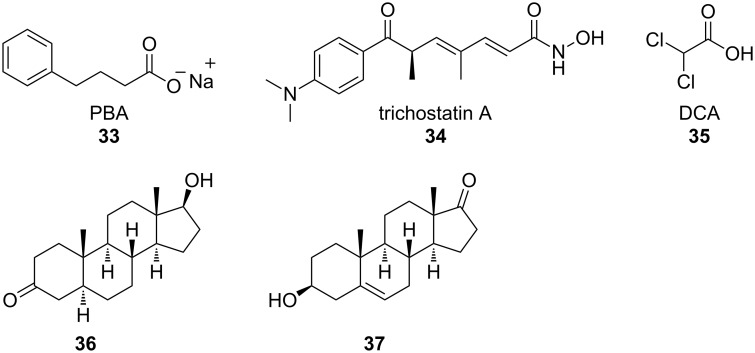
Probes used to elucidate the roles of distinct gene-expression profiles in ALS patients.

**Histone deacetylase (HDAC) inhibitors:** Several gene analysis studies have discovered distinct gene expression profiles in ALS patients [69–70], indicating that transcriptional dysfunction may contribute to ALS pathology [71]. One mechanism of eliciting changes in gene expression is through the acetylation of histone proteins, which allows access of gene sequences to transcriptional complexes. SOD1 G93A mice have markedly reduced histone acetylation following disease onset as compared to control animals [71–72], supporting a role for aberrant transcriptional activity in disease progression. Consequently, histone deacetylase (HDAC) inhibitors were tested in ALS mouse models [71–72]. Ryu et al. [71] treated SOD1 G93A mice with 400 mg/kg sodium 4-phenylbutyrate (PBA, **33**, Figure 14) by i.p. injection both prior to, and following, disease onset. Treated animals displayed increased performance on rotarod tests, improved stride length, and extended lifespan as compared to untreated animals [71]. Furthermore, astrogliosis and neuron loss in the lumbar spinal cord were attenuated with drug treatment [71], indicating that inhibition of HDACs was neuroprotective in ALS models.

Yoo et al. [72] obtained similar results using the HDAC inhibitor trichostatin A (**34**). Compound **34** was injected intraperitoneally at 1 mg/kg, 5 days a week following symptom onset in SOD1 G93A mice that had been crossed with a mouse line expressing yellow fluorescent protein (YFP) under the Thy1 promoter. These mice express YFP in motor neurons, allowing for innervation at neuromuscular junctions (NMJ) to be analyzed. Treatment with **34** increased histone acetylation in the spinal cord and skeletal muscle of SOD1 G93A mice, which corresponded with reduced motor neuron death and gliosis in the spinal cord of these animals [72]. Additionally, NMJ innervation was improved in mice treated with **34**. Behavioral testing demonstrated that rotarod performance and grip strength were improved in treated animals. Compound **34** also modestly prolonged lifespan [72].

**Glial mitochondrial function:** Although ALS is characterized by motor neuron degeneration and death, glial cells have been demonstrated to play essential roles in disease pathogenesis [73]. Inhibition of SOD1 G93A expression specifically in astrocytes increased survival in mice carrying the SOD1 G93A mutation [74], indicating the importance of glial cells to ALS progression. SOD1 mutations in astrocytes promote decreased mitochondrial function [75], an aberrant phenotype, and neurotoxicity [76]. Dichloroacetate (DCA, **35**), a compound which inhibits the pyruvate dehydrogenase enzyme, modulates mitochondrial activity [75]. Miquel et al. [75], treated SOD1 G93A mice with **35** added at 500 mg/L to their drinking water. This treatment reduced astrocyte reactivity and motor neuron death, as well as prolonged the lifespan of the treated animals by two weeks.

**Steroid treatment:** Dihydrotestosterone (DHT, **36**) treatment increases muscle mass and has been demonstrated to be neuroprotective [77]. Chronic diseases, such as ALS, which display muscle wasting may benefit from androgen treatment. To determine whether DHT treatment can ameliorate symptoms in an ALS mouse model, Yoo et al. [77] subcutaneously inserted a silastic tube containing **36** into SOD1 G93A mutant male mice at postnatal day 75. Weight and area of the gastrocnemius (GN) and tibialis anterior (TA) muscles were taken at postnatal day 120. SOD1 G93A mice treated with DHT exhibited a 32% increase in weight of the GN muscle and a 43% increase in TA muscle as compared to untreated controls [77]. Additionally, orchidectomized SOD1 G93A mice were evaluated and it was discovered that the reduced androgen concentrations in these animals exacerbated the loss in muscle weight. Cross-sectional area measurement of the GN and TA muscle displayed similar results. Compound **36**-treated SOD1 G93A mice also displayed increased muscle strength compared with untreated or orchidectomized SOD1 G93A mice, as analyzed by a grip-strength meter [77]. Interestingly, treatment with **36** increases the levels of insulin-like growth factor (IGF) 1 and 2, which induces myoblast growth and differentiation, while concomitantly decreasing the expression of muscle RING finger 1 (MuRF-1), a protein that can induce muscle atrophy [77]. The upregulation of IGF-1 and -2 and downregulation of MuRF-1 corresponded with modest increases in performance in functional tests, including the rotarod test and gait analyses. Furthermore, axonal loss and motor neuron death were slightly decreased in DHT-treated SOD1 G93A animals compared with controls [77].

However, a conflicting study has found that androgens have little effect on ALS mouse models [78]. SOD1 G93A transgenic rats were gonadectomized or treated with a neurosteroid, dehydroepiandrosterone (DHEA, **37**) prior to symptom onset. Disease progression, symptom onset, and lifespan were not affected by either **37** treatment or gonadectomy, suggesting that steroids have little effect on ALS disease pathology [78]. These conflicting results may be explained by the specific compounds that were used or the analyses that were performed. Interestingly, these studies employed different rodent models, with females being present in the DHEA study, while the DHT study was done exclusively with males. Sexual dimorphism has been previously reported in animal models of ALS, with males and females displaying differences in symptom onset and progression [78]. In ALS patients, women have fewer reported cases than men [78] suggesting that this sexual dimorphism may be replicated in humans.

## Conclusion

ALS is a complex disorder that is characterized by multiple cellular pathologies including glutamate excitotoxicity, protein aggregation, ER stress, trophic factor deregulation, oxidative stress, inflammation, and mitochondrial dysfunction. Although some cases of ALS can be attributed to known gene mutations, the cause of ALS remains largely undefined. Therefore, current treatment strategies for ALS involve the targeting of specific cellular dysfunctions.

As mutations in the SOD1 gene have been identified in 20% of fALS patients, the creation of small molecules that specifically target SOD1 has become a popular strategy for drug development. Unfortunately, due to the relatively small patient population with these specific mutations, this strategy alone may not have a large impact on ALS disease treatment. However, several new studies have focused on the prevention or reduction of SOD1 or TDP-43 aggregation. These studies may have broad applications, as mutations that lead to improper protein aggregation are a common feature in many neurological disorders including ALS [79], Charcot Marie Tooth disease [80], Alzheimer’s disease [81], and Huntington’s disease [82]. The discovery of small molecules that prevent or clear protein aggregates may prove to be valuable for the treatment of multiple disorders.

Another common strategy for the treatment of ALS is the use of compounds that elicit neuroprotection, either by upregulating molecules that promote neuronal survival or by antagonizing cellular pathways that result in neuronal death. While several of these compounds have shown promising results in SOD1 mutant animal models, it remains to be seen whether these strategies will prove effective in long-term human treatment where neurons may be exposed to multiple cellular insults.

**Figure 15:**
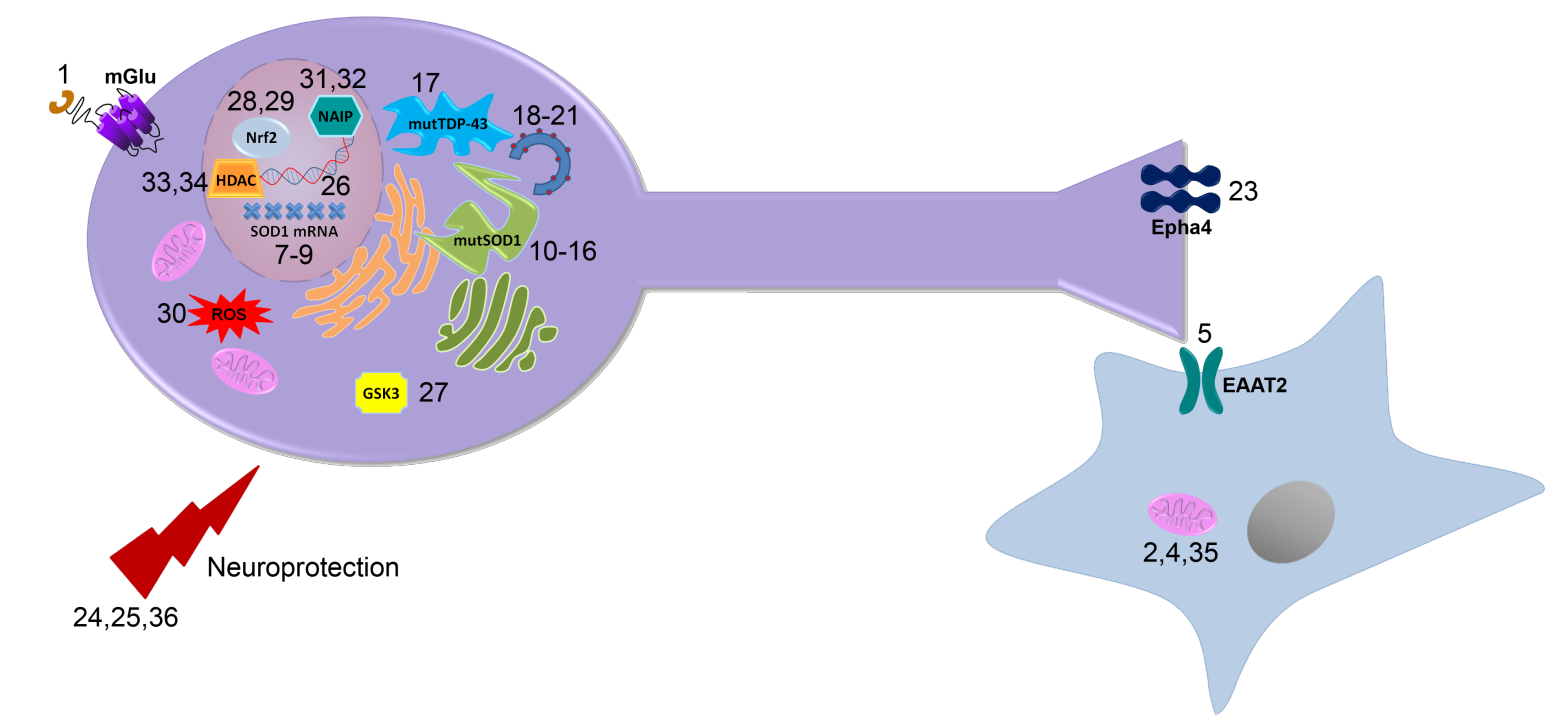
Targets of potential therapeutics: This diagram illustrates the physiological targets of each compound discussed in the review and how these compounds are believed to function in vivo.

Although progress has been made towards the development of improved ALS treatments, including several compounds in phase III clinical trials, it remains to be seen whether these treatments will prove to be efficacious in ALS patients. Various screening approaches and targeted drug design, as outlined in this review, have identified a number of small molecules that will prove useful in the discovery and validation of novel cellular targets for the treatment of ALS (Figure 15). Figure 15 illustrates the specific cellular target of each compound discussed in this review. Additionally, Table 1 in the supporting information lists each chemical structure, name, reference, and mechanism of action. Future studies toward these targets will begin to provide the necessary proof-of-concept for these alternative therapeutic approaches, lead to a greater understanding of the pathogenesis of ALS, and may lead to novel therapeutics with improved efficacy in ALS.

## Supporting Information

Supporting information features a table of each compound discussed in the review. This table contains the chemical structure, name, references and mechanism of action.

File 1 Table of compounds.

## References

[1] Pratt A J, Getzoff E D, Perry J J (2012). Degener Neurol Neuromuscul Dis.

[2] Rosen D R, Siddique T, Patterson D, Figlewicz D A, Sapp P, Hentati A, Donaldson D, Goto J, O'Regan J P, Deng H-X (1993). Nature.

[3] Glicksman M A (2011). Expert Opin Drug Discov.

[4] Contestabile A (2011). Curr Med Chem.

[5] Dunkel P, Chai C L L, Sperlágh B, Huleatt P B, Mátyus P (2012). Expert Opin Invest Drugs.

[6] Colton C K, Kong Q, Lai L, Zhu M X, Seyb K I, Cuny G D, Xian J, Glicksman M A, Glenn Lin C-L (2010). J Biomol Screen.

[7] Gurney M E, Pu H, Chiu A Y, Dal Canto M C, Polchow C Y, Alexander D D, Caliendo J, Hentati A, Kwon Y W, Deng H-X (1994). Science.

[8] Van Den Bosch L (2011). J Biomed Biotechnol.

[9] Groeneveld G J, van Kan H J M, Sastre Toraño J, Veldink J H, Guchelaar H-J, Wokke J H J, van den Berg L H (2001). J Neurol Sci.

[10] van Kan H J M, Groeneveld G J, Kalmijn S, Spieksma M, van den Berg L H, Guchelaar H J (2005). Br J Clin Pharmacol.

[11] McDonnell M E, Vera M D, Blass B E, Pelletier J C, King R C, Fernandez-Metzler C, Smith G R, Wrobel J, Chen S, Wall B A (2012). Bioorg Med Chem.

[12] Cheah B C, Vucic S, Krishnan A V, Kiernan M C (2010). Curr Med Chem.

[13] Rothstein J D, Tsai G, Kuncl R W, Clawson L, Cornblath D R, Drachman D B, Pestronk A, Stauch B L, Coyle J T (1990). Ann Neurol.

[14] Rothstein J D, Van Kammen M, Levey A I, Martin L J, Kuncl R W (1995). Ann Neurol.

[15] Danbolt N C (2001). Prog Neurobiol.

[16] Xing X, Chang L-C, Kong Q, Colton C K, Lai L, Glicksman M A, Lin C-L G, Cuny G D (2011). Bioorg Med Chem Lett.

[17] Sasabe J, Chiba T, Yamada M, Okamoto K, Nishimoto I, Matsuoka M, Aiso S (2007). EMBO J.

[18] Sasabe J, Miyoshi Y, Suzuki M, Mita M, Konno R, Matsuoka M, Hamase K, Aiso S (2012). Proc Natl Acad Sci U S A.

[19] Mitchell J, Paul P, Chen H-J, Morris A, Payling M, Falchi M, Habgood J, Panoutsou S, Winkler S, Tisato V (2010). Proc Natl Acad Sci U S A.

[20] Paul P, de Belleroche J (2012). Amino Acids.

[21] Aronica E, Catania M V, Geurts J, Yankaya B, Troost D (2001). Neuroscience.

[22] Poulopoulou C, Davaki P, Koliaraki V, Kolovou D, Markakis I, Vassilopoulos D (2005). Ann Neurol.

[23] Reaume A G, Elliott J L, Hoffman E K, Kowall N W, Ferrante R J, Siwek D F, Wilcox H M, Flood D G, Beal M F, Brown R H (1996). Nat Genet.

[24] Murakami G, Inoue H, Tsukita K, Asai Y, Amagai Y, Aiba K, Shimogawa H, Uesugi M, Nakatsuji N, Takahashi R J (2011). Biomol Screen.

[25] Wright P D, Wightman N, Huang M, Weiss A, Sapp P C, Cuny G D, Ivinson A J, Glicksman M A, Ferrante R J, Matson W (2012). Front Biosci, Elite Ed.

[26] Lange D J, Andersen P M, Remanan R, Marklund S, Benjamin D (2013). Amyotroph Lateral Scler.

[27] Wright P D, Huang M, Weiss A, Matthews J, Wightman N, Glicksman M, Brown R H (2010). Neurosci Lett.

[28] Benmohamed R, Arvanites A C, Kim J, Ferrante R J, Silverman R B, Morimoto R I, Kirsch D R (2011). Amyotroph Lateral Scler.

[29] Matsumoto G, Stojanovic A, Holmberg C I, Kim S, Morimoto R I (2005). J Cell Biol.

[30] Chen T, Benmohamed R, Arvanites A C, Ralay Ranaivo H, Morimoto R I, Ferrante R J, Watterson D M, Kirsch D R, Silverman R B (2011). Bioorg Med Chem.

[31] Chen T, Benmohamed R, Kim J, Smith K, Amante D, Morimoto R I, Kirsch D R, Ferrante R J, Silverman R B (2012). J Med Chem.

[32] Trippier P C, Benmohammed R, Kirsch D R, Silverman R B (2012). Bioorg Med Chem Lett.

[33] Zhang W, Benmohamed R, Arvanites A C, Morimoto R I, Ferrante R J, Kirsch D R, Silverman R B (2012). Bioorg Med Chem.

[34] Zhang Y, Benmohamed R, Zhang W, Kim J, Edgerly C K, Zhu Y, Morimoto R I, Ferrante R J, Kirsch D R, Silverman R B (2012). Med Chem Lett.

[35] Xia G, Benmohamed R, Kim J, Arvanites A C, Morimoto R I, Ferrante R J, Kirsch D R, Silverman R B (2011). J Med Chem.

[36] Ray S S, Nowak R J, Brown R H, Lansbury P T (2005). Proc Natl Acad Sci U S A.

[37] Nowak R J, Cuny G D, Choi S, Lansbury P T, Ray S S (2010). J Med Chem.

[38] Parker S J, Meyerowitz J, James J L, Liddell J R, Nonaka T, Hasegawa M, Kanninen K M, Lim S, Paterson B M, Donnelly P S (2012). PLoS One.

[39] Kwong L K, Neumann M, Sampathu D M, Lee V M-Y, Trojanowski J Q (2007). Acta Neuropathol.

[40] Liu-Yesucevitz L, Bilgutay A, Zhang Y-J, Vanderweyde T, Citro A, Mehta T, Zaarur N, McKee A, Bowser R, Sherman M (2010). PLoS One.

[41] Neumann M, Sampathu D M, Kwong L K, Truax A C, Micsenyi M C, Chou T T, Bruce J, Schuck T, Grossman M, Clark C M (2006). Science.

[42] Cassel J A, Blass B E, Reitz A B, Pawlyk A C (2010). J Biomol Screen.

[43] Cassel J A, McDonnell M E, Velvadapu V, Andrianov V, Reitz A B (2012). Biochimie.

[44] Suzuki H, Lee K, Matsuoka M (2011). J Biol Chem.

[45] Kim S H, Shanware N P, Bowler M J, Tibbetts R S (2010). J Biol Chem.

[46] Bose J K, Huang C-C, Shen C-K (2011). J Biol Chem.

[47] Bica L, Crouch P J, Cappai R, White A R (2009). Mol BioSyst.

[48] Wang I-F, Guo B-S, Liu Y-C, Wu C-C, Yang C-H, Tsai K-J, Shen C-K J (2012). Proc Natl Acad Sci U S A.

[49] Shimazawa M, Tanaka H, Ito Y, Morimoto N, Tsuruma K, Kadokura M, Tamura S, Inoue T, Yamada M, Takahashi H (2010). PLoS One.

[50] Zhao Z, Lange D J, Ho L, Bonini S, Shao B, Salton S R, Thomas S, Pasinetti G M (2008). Int J Med Sci.

[51] Van Hoecke A, Schoonaert L, Lemmens R, Timmers M, Staats K A, Laird A S, Peeters E, Philips T, Goris A, Dubois B (2012). Nat Med.

[52] Klein R (2009). Nat Neurosci.

[53] Pieper A A, Xie S, Capota E, Estill S J, Zhong J, Long J M, Becker G L, Huntington P, Goldman S E, Shen C-H (2010). Cell.

[54] Tesla R, Wolf H P, Xu P, Drawbridge J, Estill S J, Huntington P, McDaniel L, Knobbe W, Burket A, Tran S (2012). Proc Natl Acad Sci U S A.

[55] Feng X, Peng Y, Liu M, Cui L (2012). Neuropharmacology.

[56] Eitan E, Tichon A, Gazit A, Gitler D, Slavin S, Priel E (2012). EMBO Mol Med.

[57] Klapper W, Parwaresch R, Krupp G (2001). Mech Ageing Dev.

[58] Caporaso G L, Lim D A, Alvarez-Buylla A, Chao M V (2003). Mol Cell Neurosci.

[59] Lee J, Jo Y S, Sung Y H, Hwang I K, Kim H, Kim S-Y, Yi S S, Choi J-S, Sun W, Seong J K (2010). Neurochem Res.

[60] Lee J, Sung Y H, Cheong C, Choi Y S, Jeon H K, Sun W, Hahn W C, Ishikawa F, Lee H-W (2008). Oncogene.

[61] Hu J-H, Zhang H, Wagey R, Krieger C, Pelech S L (2003). J Neurochem.

[62] Koh S-H, Lee Y-B, Kim K S, Kim H-J, Kim M, Lee Y J, Kim J, Lee K W, Kim S H (2005). Eur J Neurosci.

[63] Koh S-H, Kim Y, Kim H Y, Hwang S, Lee C H, Kim S H (2007). Exp Neurol.

[64] Neymotin A, Calingasan N Y, Wille E, Naseri N, Petri S, Damiano M, Liby K T, Risingsong R, Sporn M, Beal M F (2011). Free Radical Biol Med.

[65] Kanno T, Tanaka K, Yanagisawa Y, Yasutake K, Hadano S, Yoshii F, Hirayama N, Ikeda J-E (2012). Free Radical Biol Med.

[66] Tanaka K, Okada Y, Kanno T, Otomo A, Yanagisawa Y, Shouguchi-Miyata J, Suga E, Kohiki E, Onoe K, Osuga H (2008). Exp Neurol.

[67] Okada Y, Sakai H, Kohiki E, Suga E, Yanagisawa Y, Tanaka K, Hadano S, Osuga H, Ikeda J-E (2005). J Cereb Blood Flow Metab.

[68] Tanaka K, Kanno T, Yanagisawa Y, Yasutake K, Hadano S, Yoshii F, Ikeda J-E (2011). Exp Neurol.

[69] Malaspina A, Kaushik N, de Belleroche J (2001). J Neurochem.

[70] Ishigaki S, Niwa J-i, Ando Y, Yoshihara T, Sawada K-i, Doyu M, Yamamoto M, Kato K, Yotsumoto Y, Sobue G (2002). FEBS Lett.

[71] Ryu H, Smith K, Camelo S I, Carreras I, Lee J, Iglesias A H, Dangond F, Cormier K A, Cudkowicz M E, Brown R H (2005). J Neurochem.

[72] Yoo Y-E, Ko C-P (2011). Exp Neurol.

[73] Boillée S, Vande Velde C, Cleveland D W (2006). Neuron.

[74] Yamanaka K, Chun S J, Boillee S, Fujimori-Tonou N, Yamashita H, Gutmann D H, Takahashi R, Misawa H, Cleveland D W (2008). Nat Neurosci.

[75] Miquel E, Cassina A, Martínez-Palma L, Bolatto C, Trías E, Gandelman M, Radi R, Barbeito L, Cassina P (2012). PLoS One.

[76] Díaz-Amarilla P, Olivera-Bravo S, Trias E, Cragnolini A, Martínez-Palma L, Cassina P, Beckman J, Barbeito L (2011). Proc Natl Acad Sci U S A.

[77] Yoo Y-E, Ko C-P (2012). PLoS One.

[78] Hayes-Punzo A, Mulcrone P, Meyer M, McHugh J, Svendsen C N, Suzuki M (2012). Amyotroph Lateral Scler.

[79] Bendotti C, Marino M, Cheroni C, Fontana E, Crippa V, Poletti A, De Biasi S (2012). Prog Neurobiol.

[80] Niemann A, Berger P, Suter U (2006). NeuroMol Med.

[81] Cavallucci V, D'Amelio M, Cecconi F (2012). Mol Neurobiol.

[82] Zheng Z, Diamond M I (2012). Prog Mol Biol Transl Sci.

